# Stromal matrix directs corneal fibroblasts to re-express keratocan after injury and transplantation

**DOI:** 10.1242/dmm.050090

**Published:** 2023-09-13

**Authors:** Ana C. Acosta, Mei Sun, Nabeel Zafrullah, Marcel Y. Avila, Curtis E. Margo, Edgar M. Espana

**Affiliations:** ^1^ Cornea and External Disease, Department of Ophthalmology, USF Health, 13330 USF Laurel Dr 4th floor, Tampa FL 33612, USA; ^2^ Universidad Nacional de Colombia, Department of Ophthalmology, Bogota 111311, Colombia; ^3^ Department of Pathology and Cellular Biology, Morsani College of Medicine, University of South Florida, Tampa, FL 33612, USA; ^4^ Department of Molecular Pharmacology and Physiology, Morsani College of Medicine, University of South Florida, FL 33612, USA

**Keywords:** Stroma, Keratocyte, Transplantation, Keratocan

## Abstract

Every tissue has an extracellular matrix (ECM) with certain properties unique to it – the tissue ‘niche’ – that are necessary for normal function. A distinct specific population of quiescent keratocan-expressing keratocytes populate the corneal stroma during homeostasis to maintain corneal function. However, during wound healing, when there is alteration of the niche conditions, keratocytes undergo apoptosis, and activated corneal fibroblasts and myofibroblasts attempt to restore tissue integrity and function. It is unknown what the fate of activated and temporary fibroblasts and myofibroblasts is after the wound healing process has resolved. In this study, we used several strategies to elucidate the cellular dynamics of corneal wound healing and the fate of corneal fibroblasts. We injured the cornea of a novel mouse model that allows cell-lineage tracing, and we transplanted a cell suspension of *in vitro*-expanded corneal fibroblasts that could be tracked after being relocated into normal stroma. These transplanted fibroblasts regained expression of keratocan *in vivo* when relocated to a normal stromal niche. These findings suggest that transformed fibroblasts maintain plasticity and can be induced to a keratocyte phenotype once relocated to an ECM with normal signaling ECM.

## Teaser

**Summary:** Cells involved in corneal stroma repair originate from corneal keratocytes. Corneal fibroblasts, originating from keratocytes, can regress to a keratocyte phenotype if relocated to a normal matrix.

## INTRODUCTION

Keratocytes, neural crest-derived cells that reside in the corneal stroma, play an important role in the acquisition and maintenance of normal stromal properties, including transparency (Espana and Birk, 2020; Fini, 1999; Poole et al., 1993). During stromal development, keratocytes regulate the synthesis and deposition of the extracellular matrix (ECM) and organize collagen fibrils (Birk and Trelstad, 1984; Young et al., 2014; Jester et al., 1994). Keratocytes are mitotically quiescent and exhibit a dendritic morphology with extensive intercellular contacts (Poole et al., 1993). They are characterized by the expression of keratocan, a proteoglycan found in the cornea exclusively in the stroma, which is thus regarded as a marker of the keratocyte phenotype (Liu et al., 1998, 2003).

During standard culture conditions on plastic dishes using fetal bovine serum (FBS), keratocytes (murine, Kawakita et al., 2005; bovine, Beales et al., 1999; rabbit, Jester et al., 1996; primate, Kawakita et al., 2006; and human, Espana et al., 2003, 2005, 2004) lose their dendritic morphology and ability to express keratocan, and become fibroblasts. These corneal-derived fibroblasts cultured at low densities or stimulated by transforming growth factor-β1 further differentiate into myofibroblasts (Jester et al., 1996; Masur et al., 1996). After injury, quiescent keratocytes are converted into fibroblasts and become mitotically active, increasing collagen and ECM synthesis and not expressing keratocan or α-smooth muscle actin (Espana and Birk, 2020; Jester et al., 1999; Wilson, 2020b). Eventually, fibroblasts can transform into α-smooth muscle actin-expressing myofibroblasts (Wilson, 2020a). Transformed fibroblasts and myofibroblasts do not express keratocan. Following resolution of wound healing, it is unknown whether fibroblasts or myofibroblasts can revert to keratocytes.

In this study, we explored the cellular dynamics that occur during wound healing and evaluate the influence of a normal ECM in regulating the conversion of fibroblasts to keratocytes.

## RESULTS

### A triple transgenic conditional *KeraRT/tetO-Cre/mTmG* mouse strain

**Fig. 1.**
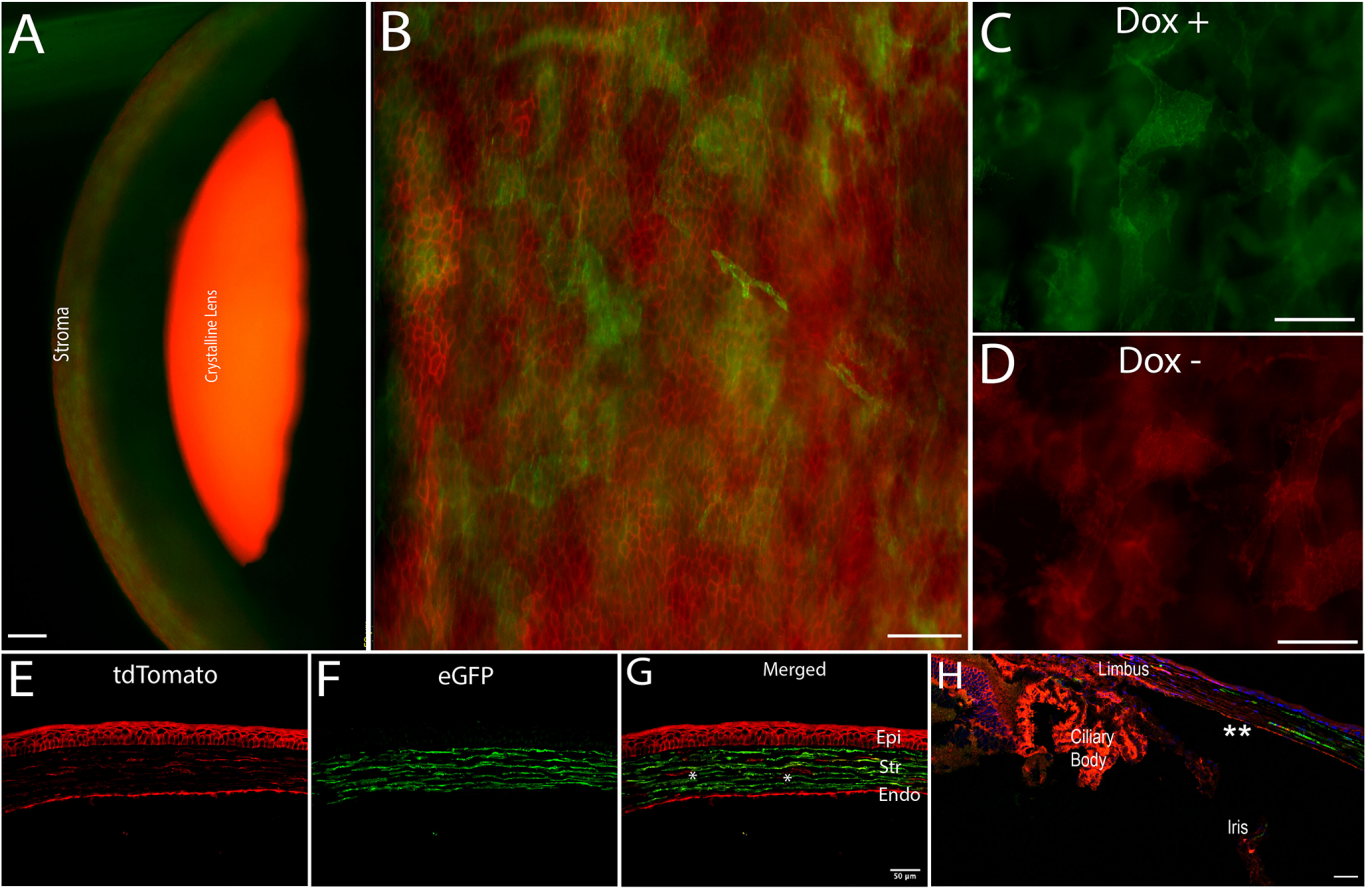
**A triple transgenic conditional *KeraRT/tetO-Cre/mTmG* mouse strain.** (A) The corneal stroma shows the presence of keratocan-expressing keratocytes (green) in the stroma of mouse eye after 1 week of oral doxycycline diet. (B) eGFP expression is limited to keratocan-expressing keratocytes only; epithelial cells do not express eGFP (green) and maintain their tdTomato (red) expression, as noted in this flat-mount image. (C,D) eGFP expression was noted in animals fed doxycycline (C) and was absent in a control group not fed a doxycycline-supplemented diet (D). (E-G) In this histology section, a few cells in the stroma did not express keratocan (marked by asterisks). The majority of cells expressed keratocan (F,G). Epi, epithelium; Str, stroma; Endo, endothelium. (H) Expression of eGFP by keratocytes started at the peripheral cornea (shown by asterisks) and was absent in the limbus. Images are representative of seven different eyes. Scale bars: 50 µm (A,E-H); 25 µm (B-D).

We created a novel conditional mouse strain to study cell lineage as well as keratocyte and fibroblast fate after injury. To evaluate whether efficient Cre excision occurred in keratocytes after 1 week with *ad libitum* doxycycline hyclate diet (Custom Animal Diets, Bangor, PA, USA), corneas were procured for examination. Under a fluorescence microscope, *KeraRT/tetO-Cre/mTmG* (I-*KeramTmG*) corneas demonstrated expression of eGFP throughout the entire stroma (Fig. 1A, showing a side view of the anterior segment). Keratocytes examined in the *z*-axis expressed eGFP (*n*=8), suggesting a high efficiency of Cre excision (Fig. 1B). eGFP expression confirmed the dendritic morphology of keratocytes within the stroma (Fig. 1C). The control group not fed with doxycycline showed no eGFP expression in any stromal or epithelial cells (Fig. 1D). Similarly, histological sections confirmed that eGFP expression was limited to stromal cells (Fig. 1E-G). Cross-section images of the cornea show how eGFP expression started at the corneal periphery and was not present in the limbal area (Fig. 1H). These analyses validated the inducible mouse model being used in the study.

### The keratocyte lineage predominates in scars after a penetrating injury in the central cornea

**Fig. 2.**
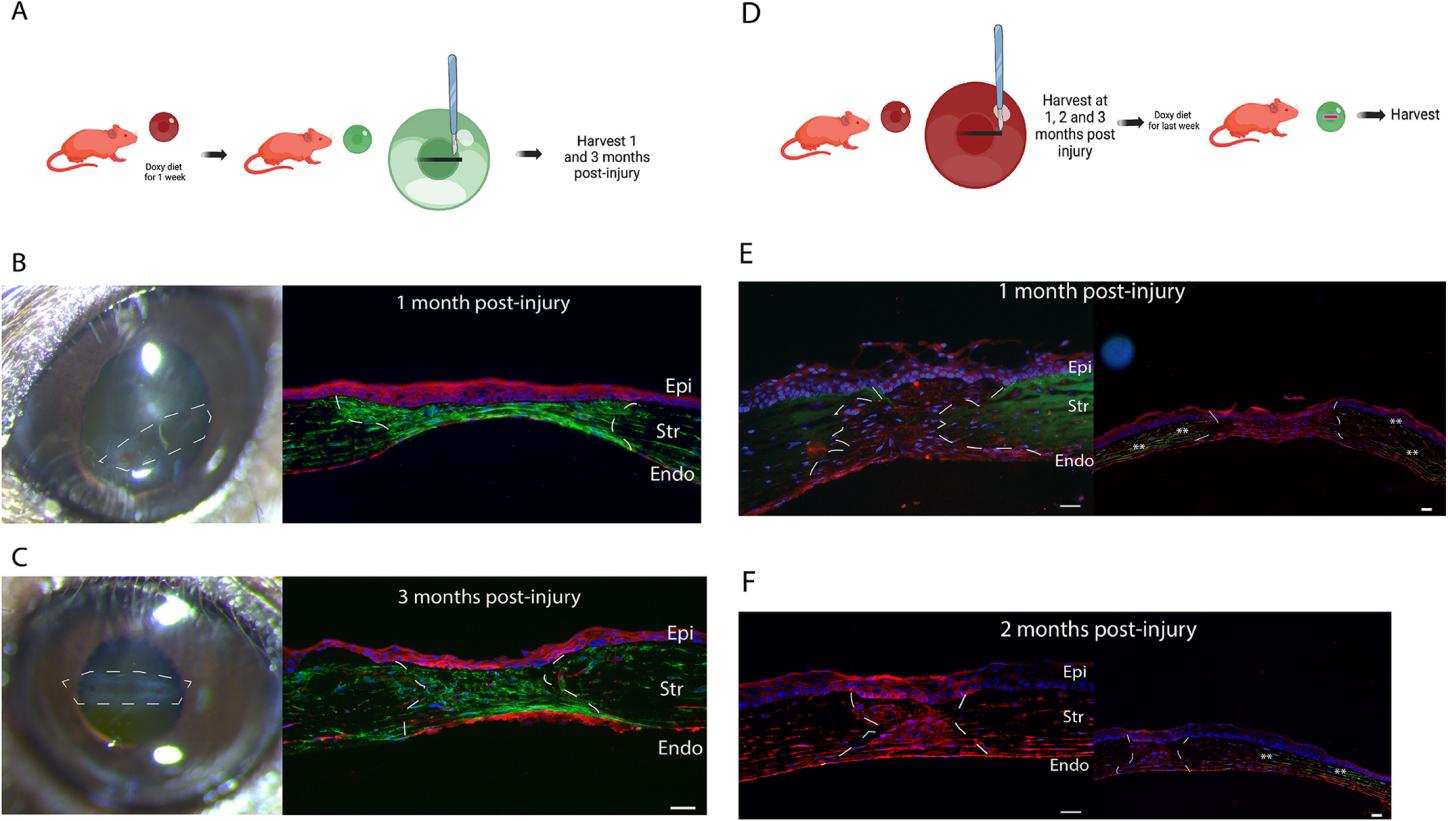
**The keratocyte lineage is involved in tissue repair post injury but stromal cells in maturing scars (up to 2 months after injury) are not keratocytes.** (A) The first strategy was to trace keratocyte lineage after the penetrating stromal injury by first labeling keratocytes with eGFP (doxycycline diet for 1 week) and then creating an injury. (B,C) Cells in the injury were derived from the keratocyte lineage as shown by eGFP expression in repaired/regenerated stroma at 1 month (B) and 3 months (C) post injury. (D) The second strategy was to evaluate the phenotype of cells present within maturing scars after the penetrating stromal injury by feeding mice a doxycycline diet for the last week before harvesting. At 1 and 2 months after the penetrating injury, cells did not express eGFP, suggesting that they were not keratocytes. (E) At 1 month post injury (*n*=6), cells present in the repaired stroma did not express keratocan. However, keratocytes were noted in the peripheral cornea outside the repaired injury area (eGFP expression shown by asterisks). (F) At 2 months after injury (*n*=6), cells present in the scar still did not express keratocan. White lines indicate scars. Images are representative of at least three experimental repeats from 16 mice. Epi, epithelium; Str, stroma; Endo, endothelium. Scale bars: 25 µm (B,C, right; E,F).

As keratocan in the cornea is exclusively expressed by keratocytes and the expression of eGFP by Cre recombinase in our mutant model is maintained in cell progeny once activated, the expression of eGFP represents a true fate map and strongly suggests that cells in maturing and matured scars after a full-thickness laceration are derived from keratocytes. We created a full-thickness wound and studied the phenotype of cells present in maturing scars (1-2 months after injury) and mature scars (3 months after injury) (Fig. 2A). Evaluation of multiple samples by immunofluorescence microscopy at 1 (*n*=6) and 3 months (*n*=6) showed that all cells in scars expressed eGFP, demonstrating their keratocyte lineage. Fig. 2B shows a representative sample collected 1 month after injury and Fig. 2C shows a representative sample 3 months post injury. Rarely, tdTomato-expressing cells were found in scars. These findings suggest that cells derived from keratocytes are responsible for repair and regeneration of stromal injuries. For the first time, this is demonstrated in an *in vivo* injury model.

### Maturing scars do not harbor keratocytes and are populated by keratocyte-derived stromal cells

Corneal scars are graded along a 4-point scale with 3 being the worst (Cogswell et al., 2021). We decided to focus our study on scars with grades 1 and 2 and avoid severe scars with significant vascularization, matrix disorganization and infiltration of CD68^+^ lineage cells (Cogswell et al., 2021). It is believed that keratocytes repopulate areas of injury during scar maturation (Wilson, 2020b) and it is unknown whether fibroblasts and myofibroblasts present in scars undergo apoptosis or regress to a keratocyte phenotype. To evaluate whether keratocytes are present in maturing scars, we used a strategy in which we created scars using our full-thickness wound injury model and, 1 and 2 months later, we induced eGFP expression by Cre recombinase activation by feeding animals with doxycycline for the last week before harvest. Collagen fibril labeling – by applying 5[(4,6-dichlorotriazin-2yl)-amino]fluorescein (DTAF) at the time of surgical procedure – was used to distinguish collagen in the regenerated matrix (unlabeled) from old (fluorescent and DTAF-labeled) collagen (Fig. 2D). We found no eGFP-expressing cells – suggesting that no keratocytes are present – in maturing scars 1 month after injury (*n*=6) (Fig. 2E). Similarly, immunofluorescence microscopy using a primary antibody against keratocan showed no keratocan expression in the regenerated stromal matrix 1 month post injury (Fig. S1B). We also could not identify keratocytes when 2-month-old maturing scars were studied (*n*=6) (Fig. 2F). In contrast, eGFP-expressing cells were abundant in the cornea surrounding the scar and in peripheral uninjured cornea (Fig. 2E,F, right). These findings suggest that keratocytes are not able to invade scars 2 months after injury from the peripheral cornea or that fibroblasts in a maturing scar have not regressed to keratocytes. We could not rule out that the doxycycline could not penetrate the core of maturing scars due to fibrosis and failed to activate eGFP expression.

### Lack of spatial organization of stromal cells and regenerated matrix in mature scars 3 months post injury

**Fig. 3.**
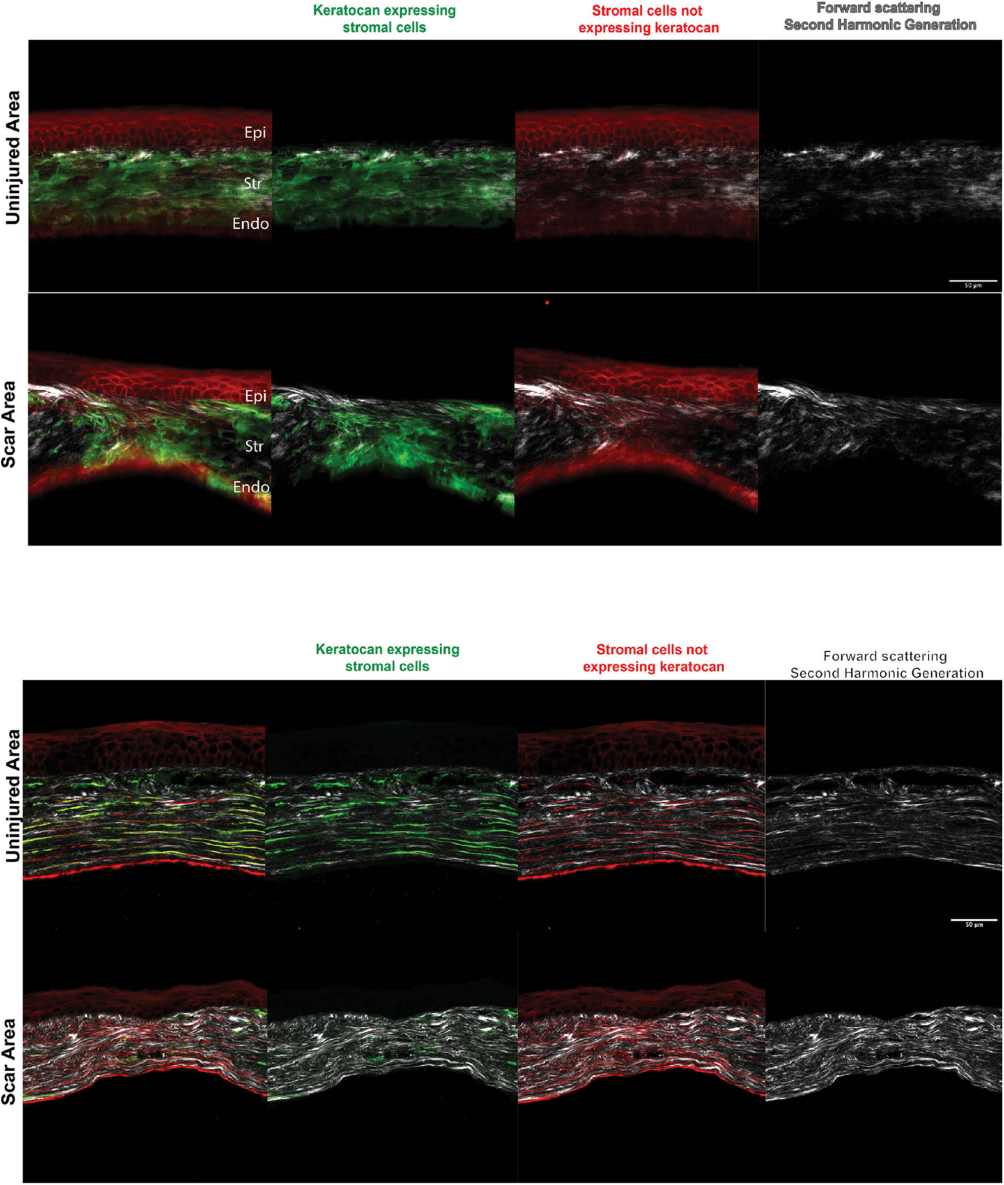
**Imaging of mature corneal scars using SHG and fluorescence microscopy in a freshly enucleated adult I-*KeramTmG* eye mounted for imaging shows keratocytes in the repairing stroma 3 months after injury.** A doxycycline diet was given 1 week prior to injury to assess eGFP expression in scars. Top: uninjured corneas had a predominance of keratocytes embedded within the corneal stroma, with few cells expressing tdTomato. Second-harmonic generation (SHG) imaging (gray, right) showed horizontally well-aligned and organized collagen fibrils/lamellae in the stroma. The scar area showed a mixture of keratocytes and tdTomato-expressing cells in the scar tissue, which was disorganized in an oblique and vertical array. Similarly, collagen fibrils/lamellae were disorganized (*n*=4). Bottom: tissue sections showed keratocytes in the repairing stroma 3 months after injury. Uninjured corneas had a predominance of keratocytes embedded within the corneal stroma (green and red signals), with a minimal number of cells expressing tdTomato (red). Cells were parallel to the basement and Descemet's membranes. SHG imaging (gray, right) showed well-aligned and horizontally organized collagen stromal fibrils/lamellae. Scars had a mixture of keratocytes and tdTomato-expressing cells oriented in a disorganized fashion. Similarly, collagen fibrils/lamellae were disorganized (*n*=6). Epi, epithelium; Str, stroma; Endo, endothelium. Scale bars: 50 µm.

To study the organization and phenotype of cells (eGFP-expressing or not expressing eGFP) and that of the deposited collagen matrix in mature corneal scars (arbitrarily defined by us as scars formed at 3 months after injury), we used immunofluorescence and second-harmonic generation (SHG) imaging microscopy for freshly mounted globes and histology sections (Fig. 3, top and bottom, respectively). Animals were fed doxyxycline 1 week before harvesting to induce eGFP expression. We found a mixed population of stromal cells – eGFP-expressing keratocytes and stromal cells not expressing keratocan in mature scars. Keratocytes were noted in all samples studied. No clear cell polarity or network organization was seen, and they were not aligned parallel to the epithelial layer or Descemet's membrane as they are in normal uninjured controls. Cells in the scar displayed random orientation in relation to cells in the epithelial layer and Descemet's membrane. Keratocytes were decreased or absent in areas of disorganized stroma (scars). SHG imaging showed a poor arrangement and orientation of the collagen matrix deposited by the cells in the regenerated matrix in comparison to that in uninjured normal controls. Taken together, these data show that scars consist of a disorganized ECM deposited by stromal cells in disarray and that keratocytes are present in mature scars.

### Keratocan expression is lost during standard culture conditions and is regulated by matrix stiffness *in vitro*

**Fig. 4.**
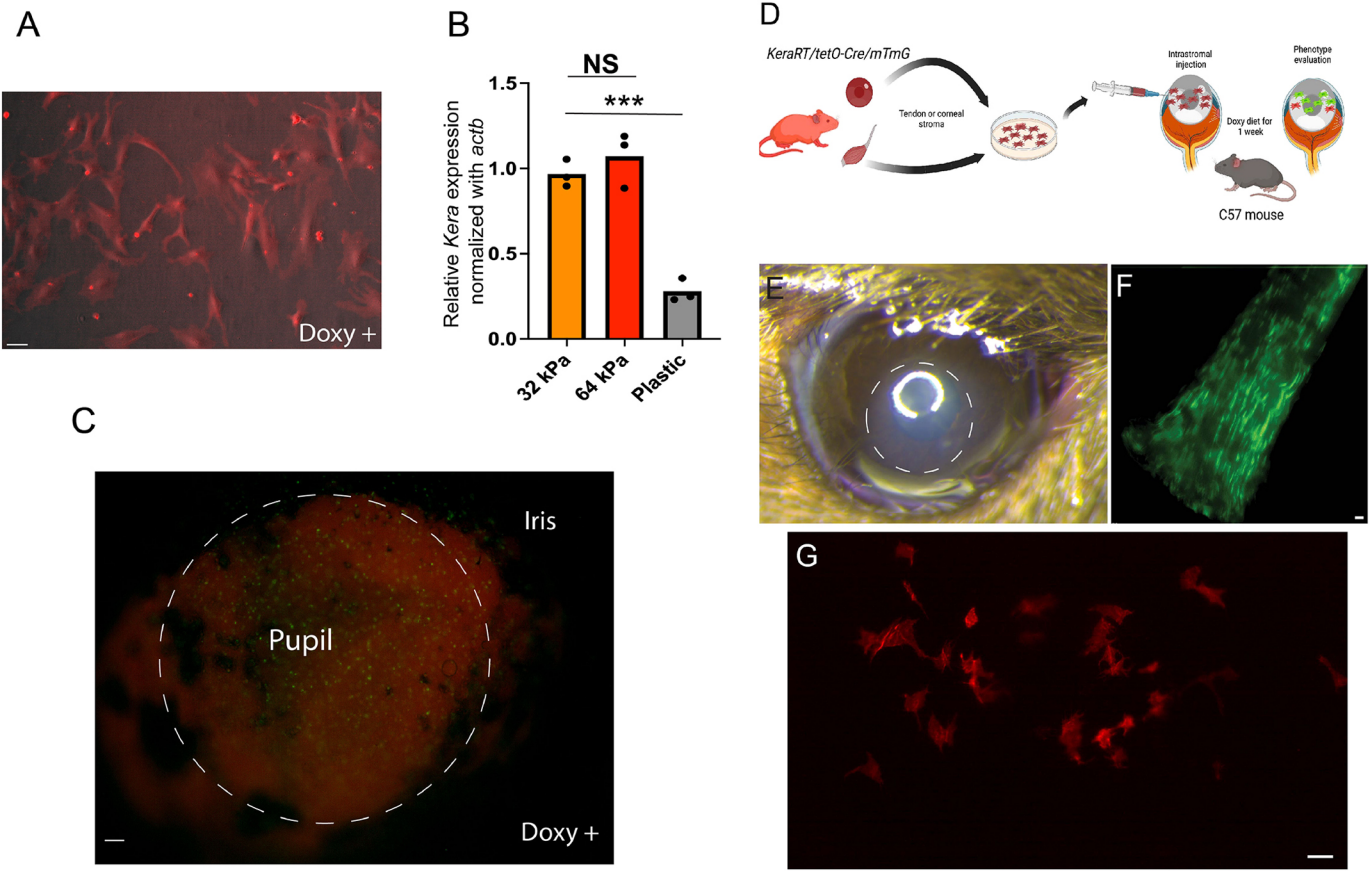
**Keratocan expression is lost during standard *in vitro* expansion in plastic culture dishes with serum stimulation but maintained or reacquired if cells are in their unique stromal niche.** (A) Freshly passed fibroblasts expanded *in vitro* under serum stimulation shut down keratocan expression, despite maintaining a dendritic morphology (*n*=3). (B) Stromal cells downregulated keratocan expression when cultured in a stiffer matrix; plastic dishes in this case (*n*=3). In contrast, keratocytes expressed eGFP *ex vivo* – in serum medium supplemented with doxycycline – when maintained within the stromal matrix. NS, not significant; ***P=0.0003 (two-tailed unpaired *t*-test). (C) The image shows induced cells (eGFP expression) that are more noticeable in the region of the pupil 24 h after exposure to doxycycline in the culture medium (*n*=5). (D) Expanded tenocytes – known to express keratocan *in vivo* – or corneal-derived fibroblasts can be expanded *in vitro* and transplanted to the stromal matrix to evaluate whether keratocan is expressed. (E) An area of corneal edema (dashed circle) developed immediately after cell injection; the edema resolved in a few days and corneal transparency returned to normal (*n*=7). (F) eGFP expression in tendons *in vivo* after 1 week of oral doxycycline diet (*n*=6). (G) However, expanded tenocytes failed to express keratocan (eGFP) when transplanted to the stromal matrix (*n*=5). Scale bars: 25 µm (A,C,F,G).

Corneal keratocytes cultured on plastic dishes under serum stimulation lose their dendritic morphology and fail to express keratocan (Kawakita et al., 2005; Espana et al., 2003). To confirm that our expanded I-*KeramTmG* cells lost expression of keratocan, we expanded freshly isolated stromal I-*KeramTmG* keratocytes *in vitro*. Fluorescence phase-contrast microscopy demonstrated no eGFP expression with doxycycline in 5% serum-supplemented medium when cells were expanded on plastic, as early as the first cell passage. Not a single cell fluoresced green after the first passage after culture in medium containing doxycycline (Fig. 4A). Interestingly, when stromal cells were cultured on a matrix that was similar in stiffness to the normal stroma (30-60 kPa; Sun et al., 2020b), keratocan mRNA expression was higher compared to that in stromal cells cultured on plastic (with significantly higher stiffness) (Fig. 4B). Once we determined that matrix stiffness was a factor regulating keratocyte phenotype, we wanted to study whether keratocan expression was maintained when cells were kept in their normal environment and exposed to serum. For this purpose, freshly isolated corneas with the epithelial layer enzymatically removed with dispase in 5% FBS and Dulbecco's modified Eagle medium (DMEM) were exposed to doxycycline for 24 h. A great majority of stromal cells expressed eGFP (Fig. 4C).

### Only early-passage corneal fibroblasts that were transplanted into a normal corneal matrix regain keratocan expression

**Fig. 5.**
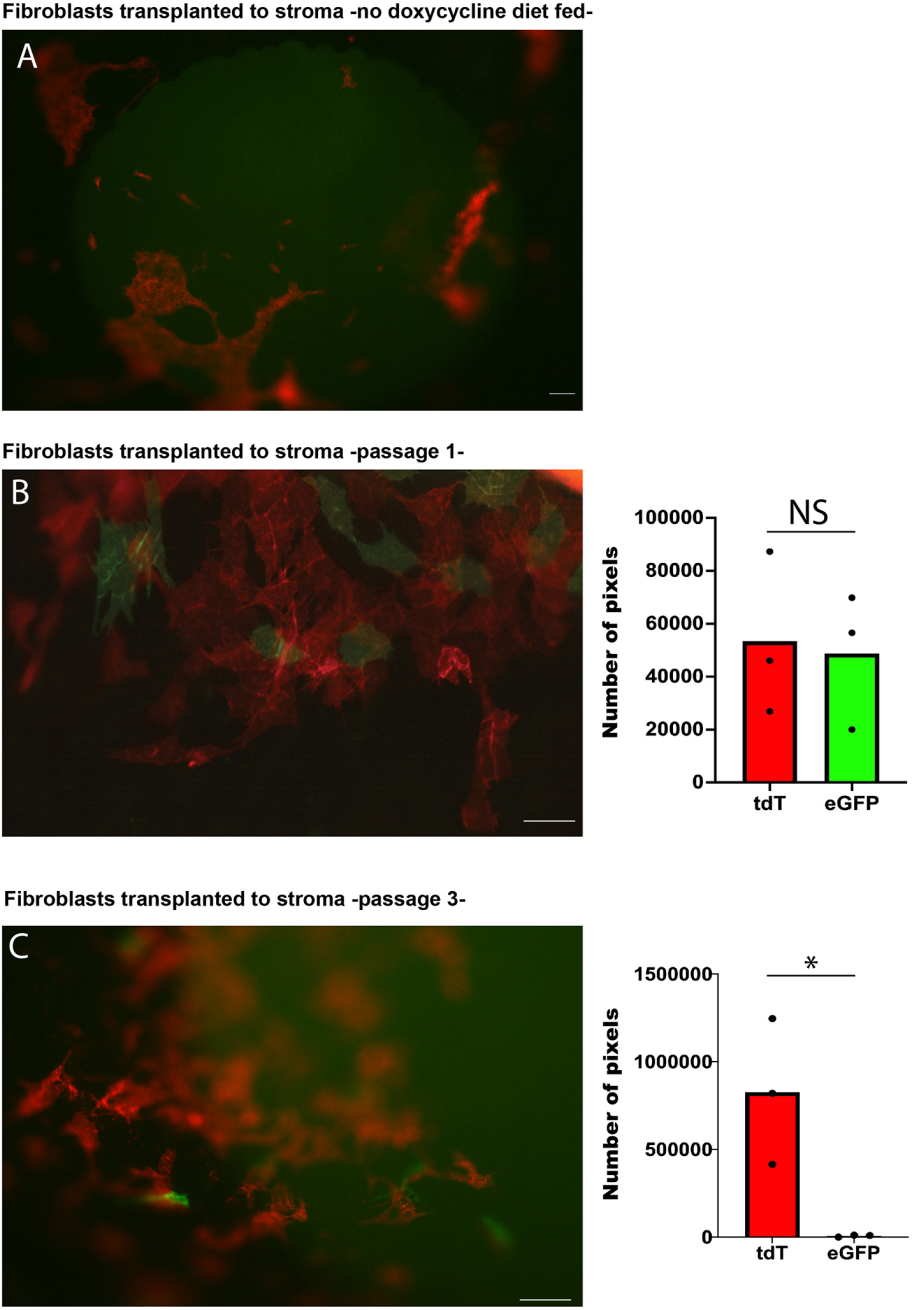
**Fibroblasts revert to a keratocyte phenotype after transplantation.** (A) Transplanted cells in mice not fed with a doxycycline-supplemented diet did not express eGFP. (B) High-magnification microscopy shows the presence of keratocytes (eGFP-expressing cells) 1 week after transplantation into a normal stromal niche in mice fed with a doxycycline-supplemented diet (passage 1 cells) (*n*=9). (C) Very few cells expressed keratocan once corneal fibroblasts were expanded for a longer time *in vitro* (passage 3) (*n*=3). The graphs (right) show quantification of the number of image pixels for tdTomato (tdT)- and eGFP-expressing cells. NS, not significant; *P<0.05 (two-tailed paired *t*-test). Scale bars: 50 µm.

To explore whether the altered properties of scar matrix limit fibroblast conversion to keratocytes, we used a modified surgical technique for relocation of corneal fibroblasts into normal stroma (Call et al., 2019; Ghoubay et al., 2020). *In vitro*-expanded fibroblasts or tenocytes derived from I-*KeramTmG* mice were injected into corneas of adult wild-type (WT) mice (Fig. 4D). Corneal swelling was noted in the area immediately after the injection (Fig. 4E) but resolved 2-3 days after injection. WT mice were given 7 days of doxycycline-supplemented diet *ad libitum* to allow Cre recombinase-dependent eGFP expression in the relocated I-*KeramTmG* fibroblasts or tenocytes. Examination under fluorescence microscopy showed successful transplantation and survival of transplanted cells in the stroma of WT mice. Different controls were used in these experiments. Expanded tenocytes known to express keratocan *in vivo* (Fig. 4F) failed to express keratocan after transplantation to the stroma of WT host (Fig. 4G). Transplanted corneal fibroblasts into normal WT stromal host failed to express eGFP if not fed with doxycycline-supplemented diet (Fig. 5A). However, expanded fibroblasts transplanted to WT hosts and fed with a doxycycline-supplemented diet for 1 week consistently showed the presence of eGFP. We found no statistically significant difference in the proportion of cells that expressed eGFP and tdTomato, suggesting that a good proportion of fibroblasts regressed to keratocytes (Fig. 5B). We consistently noticed eGFP-expressing keratocytes when *in vitro*-expanded fibroblasts up to passage 3 were transplanted. However, fewer cells expressed eGFP after induction when passage 3 cells were transplanted. A great majority of cells maintained tdTomato expression (Fig. 5C). There was a statistically significant difference between eGFP- and tdTomato-expressing cells when passage 3 cells were transplanted into a WT host. These data suggest that fibroblasts in a normal stromal niche, under normal conditions, can reverse their phenotype to cornea-specific keratocytes.

## DISCUSSION

The origin and fate of fibroblasts following corneal injury is not firmly established. It has yet to be determined if they can revert to keratocytes *in vivo*. To confound matters, recent evidence suggests that bone marrow-derived cells may also participate in the wound-healing process, and some could differentiate to myofibroblasts (Wilson, 2020b; Saikia et al., 2020; Wilson et al., 2021). In this study, we demonstrate for the first time that, *in vivo*, stromal cells in corneal scars are derived from keratocytes and that fibroblasts can revert back to keratocytes if a healthy stromal matrix allows it.

Understanding the lineage of origin and fate of stromal cells involved in tissue repair has translational implications, including therapeutical opportunities to modulate wound healing and mitigate stromal scarring. Cell-lineage tracing using fluorescent reporter mouse models has helped to determine the cell of origin of fibroblasts and myofibroblasts in different tissues and organs. Tissue-resident fibroblasts, pericytes, epithelial cells, fibrocytes and bone marrow-derived mesenchymal stem cells are known to become repairing fibroblasts or myofibroblasts once activated in multiple organs (Pakshir et al., 2020). The I-*KeramTmG* mouse model is a tool designed to address these questions in the corneal stroma *in vivo*. We found that all cells in the repaired stroma expressed eGFP, suggesting that they originated from a keratocyte lineage. These findings confirm that keratocytes are the source of cells residing in scars. However, we could not exclude that bone marrow-derived cells played a role in tissue repair.

Our I-*KeramTmG* mouse model gave us some answers on the fate of corneal fibroblasts during tissue repair and in mature scars. The process of wound healing is complex and affected by three main players: cellular agents, the ECM and the immune system. The fate of fibroblasts and myofibroblasts once tissue repair advances is unknown, but the options are limited: they either die (apoptosis) or regress to keratocytes (Wilson, 2020a; Pakshir et al., 2020; Schuster et al., 2023). Senescence of myofibroblasts – an alternative fate – has been discussed as a mechanism promoting tissue fibrosis (Merkt et al., 2021; Hinz and Lagares, 2020). The possibility that fibroblasts reverse to a keratocyte phenotype has intrigued corneal biologists for years. Does a successfully repaired matrix direct the return of fibroblasts to keratocytes, or is the invasion of keratocytes – and probable repairing function – from the surrounding tissue to scars necessary to restore function, including transparency? We used two different strategies to study fibroblast-to-keratocyte reversibility. First, we evaluated keratocan expression in cells present in the repaired matrix and scars, up to 3 months after injury, by inducing expression of eGFP by Cre recombinase. By studying eGFP expression in maturing and mature scars, we could determine whether cells were keratocytes. We found no eGFP expression at 1 month after injury but different degrees of eGFP expression were observed in mature scars (3 months after injury). These findings demonstrate that scar maturation is a dynamic process and that the phenotype of cells within scars change with time. What these changes in cell phenotype mean is unknown and their implications speculative.

Our data show that eGFP expression *ex vivo* by Cre recombinase can be induced if cells are within a normal stroma. eGFP expression does not occur when cells – even at an early passage – are expanded *in vitro* in standard culture conditions. Knowing what factors direct the transition of cells back to keratocytes in corneal scars is crucial to modulate and manipulate corneal wound healing. We found through *in vitro* and *ex vivo* studies that ECM properties are in part responsible for the maintenance of the keratocyte phenotype. During wound healing, the ECM is critical to physically support tissues and to regulate the availability of different growth factors and modulate cytokine availability, e.g. decorin overexpression has been shown to decrease corneal scars density (Mohan et al., 2011a,b, 2022). Although multiple factors could regulate the phenotype and activation of keratocytes, matrix stiffness, at least *in vitro*, was found to regulate the expression of keratocan. Stromal cells expanded on a matrix with normal stiffness did not exhibit a reduction in keratocan mRNA expression as much as cells expanded on a rigid traditional plastic culture dish. It is well known that ECM stiffness regulates cell function and that mechanotransduction is essential in regulating multiple intracellular pathways (Humphrey et al., 2014). Multiple cell mechanisms are upregulated in a stiff matrix (dense scar) compared to a normal matrix. More intracellular pathways are activated in a stiffer matrix with increased recruitment of proteins to focal adhesions (Henderson et al., 2020). Besides, the activation of latent growth factors is also upregulated in a stiff matrix (Hinz, 2009, 2015). A stiff matrix will facilitate the activation of fibroblasts, and, therefore, deposition of an abnormal disorganized matrix and fibrosis with persistent loss of corneal functions, including transparency.

The second strategy we used was to show the importance of a normal matrix in directing regression of fibroblasts to keratocytes: the technique of relocating fibroblasts – not expressing keratocan, in a state of activation with active mitoses and in the expansion phase after being transformed *in vitro* using serum stimulation – into normal adult stroma and then inducing the expression of eGFP by Cre recombinase. Using this technique, we demonstrated that a healthy stromal matrix controls the fate of fibroblasts and keratocytes. This is of translational significance and opens doors to explore the use of decellularized corneal tissue for transplantation, i.e. the effects of the decellularization process in the ultrastructure, biomechanical and biological properties of the tissue will be crucial for success (Polisetti et al., 2021).

We have, for the first time, demonstrated *in vivo* that activated corneal fibroblasts, or a significant percentage of them, return to a quiescent keratocyte phenotype when placed in a normal corneal microenvironment (the quiescent stromal niche). The ECM specific to each tissue in the body has unique signaling and functions but, clearly, in addition to ECM signaling, tissue mechanics are also important for proper tissue function. Cell-specific factors are crucial. This is demonstrated when tenocytes expanded *in vitro* failed to reattain expression of keratocan after being relocated to the corneal milieu. Similarly, we do not know why some corneal fibroblasts revert to a keratocyte phenotype, whereas others do not, but early passage-1 cells expressed much more keratocan than passage-3 fibroblasts. Preliminary data in our laboratory show no reversal of expanded myofibroblasts to keratocytes. Mechanical memory and epigenetic changes could explain these findings.

Being able to manipulate corneal fibroblasts and return them to a keratocyte phenotype is of utmost importance. Now that we have demonstrated that fibroblasts are endowed with phenotype plasticity, relevant clinical questions can be addressed. We have started by studying the role of matrix disorganization in fibroblast plasticity by using SHG imaging. We believe that matrix disorganization – present in corneal scars – challenges the reversal of fibroblasts to a keratocyte phenotype. The importance of persistent survival of activated fibroblasts and myofibroblasts in maintaining this matrix disorganization is unknown. Therapies promoting myofibroblast deactivation – apoptosis, reprogramming and clearance of senescent myofibroblasts – are of interest. Future studies should determine the influence of manipulating growth factors and applying gene therapy and chemical pathway inhibitors in facilitating the reversibility of fibroblasts to keratocytes by regulating mechanotransduction *in vivo*. Our laboratory is actively working in manipulating matrix stiffness by downregulating the expression of different collagens during scar maturation. We believe that manipulating matrix stiffness during wound healing can indirectly regulate mechanotransduction.

Finally, the findings in this study also show the feasibility of transplanting genetically modified cells that can be altered (induced) in the recipient tissue for therapeutic benefit. Opportunities that this technique can potentially provide include cell therapy for regeneration of damaged tissue (stromal dystrophies). This technique also has the advantage that gene induction can be temporally and spatially controlled. Future studies in larger animals are needed to confirm our findings. In conclusion, we have demonstrated that keratocyte progeny oversees regenerating stromal tissue and repairing matrix defects. The influence that the matrix has on keratocyte phenotype and the reversibility of fibroblasts to a keratocyte phenotype during tissue repair provides a window of opportunity for therapeutic manipulation and amelioration of corneal scarring following injury.

## MATERIALS AND METHODS

### Animals

To study cell dynamics during corneal injury and trace the fate of expanded fibroblasts and their conversion to keratocytes, an inducible *KeraRT/tetO-Cre* mouse model obtained from Prof. Chia-Yang Liu’s laboratory (Department of Ophthalmology, University of Cincinnati) (Zhang et al., 2017) was bred with the *Rosa26^mTmG^* mouse (stock 008463, The Jackson Laboratory, Bar Harbor, ME, USA). The *mTmG* mouse strain is a double-fluorescent Cre reporter mouse strain that expresses the membrane-targeted tandem-dimer Tomato (mT) ubiquitously prior to Cre-mediated excision. An inducible triple transgenic conditional *KeraRT/tetO-Cre/mTmG* mouse strain, which we named I-*KeramTmG*, was generated*.* Enhanced green fluorescent protein (mG) is expressed after Cre excision (Muzumdar et al., 2007) and, in the case of I-*KeramTmG*, eGFP was only present in cells expressing keratocan after animals were fed oral doxycycline for 1 week*.* Adult (60-day-old) WT C57BL/6 mice were used for transplantation studies, evaluating the differentiation of I-*KeramTmG* corneal-derived fibroblasts (tdTomato-expressing, red) to keratocytes (eGFP-expressing, green) if relocated to a normal stroma.

### Creation of a full-thickness keratotomy for evaluation of stromal cell phenotype dynamics during wound healing

For injury experiments, adult 60-day-old male mice were anesthetized with intraperitoneal injection of ketamine (100 mg/kg; Mylan Pharmaceuticals, Rockford, IL, USA) and xylazine (10 mg/kg; Mylan Pharmaceuticals), and subcutaneous analgesia was administered as described above. Once the mouse was under general anesthesia, 0.1 ml of 0.5% proparacaine hydrochloride (Alcon Labs, Dallas, TX, USA) and 1% atropine (Alcon Labs) were placed on the ocular surface. By visualization under a microscope, a full-thickness corneal incision, 1 mm in length, was made centrally in the cornea with a 15-degree blade. The stromal matrix was labeled with 5[(4,6-dichlorotriazin-2yl)-amino]fluorescein (DTAF, Sigma-Aldrich) in six I-*KeramTmG* corneas to demarcate the areas of tissue repair (Sun et al., 2020a). Immediately after the procedure, drops of moxifloxacin (Alcon Labs) and artificial tear ointment (Alcon Labs) were applied to the ocular surface. Mice were housed and treated in accordance with the National Institutes of Health's Guide for the Care and Use of Laboratory Animals. Eyes were evaluated at 4, 9 and 14 weeks after injury.

### Isolation and culture of corneal fibroblasts

After euthanasia, the eyes of adult 60-day-old *I-KeramTmG* mice were enucleated and copiously washed with betadine ophthalmic solution (Alcon, Fort Worth, TX, USA). Eyes were incubated in DMEM (Thermo Fisher Scientific) containing 15 mg/ml dispase II (Roche Applied Science) at 4°C for 18 h. The entire corneal epithelium could then be removed by vigorous shaking. Under a dissecting microscope, the corneal stroma was separated from the sclera at the limbus by pressing down the corneoscleral junction with a 27-gauge needle (Kawakita et al., 2005). Isolated corneal stromas were incubated overnight at 37°C in DMEM containing 1.25 mg/ml collagenase A (Roche Applied Science, Penzberg, Germany) and 25 μg/ml gentamicin (Sigma-Aldrich). A keratocyte-containing cell suspension was initially seeded on 24-well dishes and then passed to T25 flasks (Thermo Fisher Scientific) in DMEM containing ITS [5 μg/ml insulin (Thermo Fisher Scientific), 5 μg/ml transferrin (Thermo Fisher Scientific) and 5 ng/ml sodium selenite (Thermo Fisher Scientific)], and 25 μg/ml gentamicin supplemented with 5% FBS (Thermo Fisher Scientific). The suspension of keratocytes prepared from six mouse corneal buttons was poured into each flask. *In vitro-*expanded fibroblasts and freshly procured corneal tissue was cultured with medium containing 1000 ng/ml doxycycline hyclate (Sigma-Aldrich, D9891) to induce eGFP expression *in vitro*. Cells were passed in a 1:3 ratio at 80% confluence. Tenocytes obtained from *I-KeramTmG* mice were isolated and expanded as previously reported (Smith et al., 2014).

### RNA isolation and quantification of mRNA in expanded corneal fibroblasts

Total RNA was extracted from expanded fibroblasts using QIAzol lysis reagent (QIAGEN, Venlo, Limburg, the Netherlands) and RNeasy MinElute Cleanup Kit (QIAGEN). Reverse transcription and quantitative real-time PCR analysis was performed as described before (Sun et al., 2020b). The following primer sequences were used: *Keratocan*: forward, 5'-CCTGGAAAGCAAGGTGCTGTA-3', and reverse, 5'-TCATAGGCCTGTCTCACACTCTGT-3'; *Actb*: forward, 5'-AGATGACCCAGATCATGTTTGAGA-3', and reverse, 5'-CACAGCCTGGATGGCTACGT-3'. Each PCR sample was run in duplicate, and statistical analysis was performed after experiments were repeated at least three times.

### Corneal fibroblast transplantation model

Animals were anesthetized with intraperitoneal injection of ketamine (100 mg/kg) and xylazine (10 mg/kg). Only left corneas were subjected to cell transplantation. By visualization under a microscope, special 33-gauge beveled needles with an angle of 12 degrees and a length of 10 mm together with a 50 µl glass syringe were used (Hamilton Company, Reno, NV, USA). Needles were inserted under microscopic visualization into the mid-corneal stroma and beveled laterally during injection of the cell suspension. A mild diffuse intrastromal haze was noted when an injection was successfully performed. Adult 60-day-old WT mice were used as recipients. A total of 10 µl with approximately 5000 cells was injected into each cornea. Experiments were performed in accordance with the Institutional Animal Care and Use Committee research protocol number IS00010330.

### Immunofluorescence and SHG microscopy

Fresh eyes were procured from adult I-*KeramTmG* and WT mice after transplantation for histological and SHG imaging and evaluation. Fresh eyes were harvested and embedded in optimum cutting temperature (OCT) medium (TissueTek, Sakura, Torrance, CA, USA) and frozen with isopentane (Sigma-Aldrich) on dry ice. Unfixed 7 µm-thick corneal sections (NX 50 cryostat, Thermo Fisher Scientific) were covered with Vectashield mounting solution (Vector Laboratories, Burlingame, CA, USA), with DAPI used as a cell nuclei marker. Some sections were stained using an antibody against keratocan (kind gift from Dr Chia-Yang Liu, 1:100 dilution of primary antibody) (Zhang et al., 2017). Images were captured using a fluorescence microscope (Leica, DM5500B). Identical conditions and negative controls facilitated comparisons between samples. Images of corneal tissue, *en face*, using *z*-stacks were also captured using the same microscope. For SHG imaging, enucleated eyes were immediately placed in Optisol medium (Bausch and Lomb, Bridgewater, NJ, USA) on a custom-made glass chamber and imaged (within 5 min) without any tissue manipulation or additional dissection. Corneal cross-sections were imaged using an Olympus MPE-RS microscope using a 25× (0.95 NA) water-immersion objective (Olympus corporation, Tokyo, Japan). Two-photon SHG signals were generated using a mode-locked titanium:sapphire laser at 960 nm. The SHG forward-scattered signals passing through the corneal sections were collected using a 0.8 NA condenser lens with a narrow band-pass filter (465-485 nm). All samples were scanned using a 2 μm *z*-axis step size from the back to the front of the section.

### Quantification of keratocytes and fibroblasts following fibroblast transplantation to the corneal stroma

Image sets captured from videos obtained by fluorescence microscopy were opened in ImageJ (National Institutes of Health, Bethesda, MD, USA) software and scaled to micrometers. Thereafter, the color thresholding function was used to isolate areas of green versus red intensity. The total area of each of these colors was measured and calculated using the measure function for the total thresholded area that corresponded to the desired color (red or green). These values were then compared for the passage 1 (P1) and 3 (P3) sets.

### Statistical analysis

GraphPad Prism version 9.1.2 (San Diego, CA, USA) was used for statistical analyses and all data are shown as means±s.d. Statistical significance between two conditions was evaluated by unpaired two tailed Student’s *t*-test. Values with *P*<0.05 were considered statistically significant (*P<0.05; **P<0.01; ***P<0.001; ****P<0.001).

## Supplementary Material

10.1242/dmm.050090_sup1 Supplementary information
