# Supplementary material for: Stromal matrix directs corneal fibroblasts to re-express keratocan after injury and transplantation
Source: Dis Model Mech. 2023 Sep 13;16(9):dmm050090. doi: 10.1242/dmm.050090 (PMC10508697; doi:10.1242/dmm.050090)
Supplement: Supplementary information [file dmm-16-050090-s1.pdf]

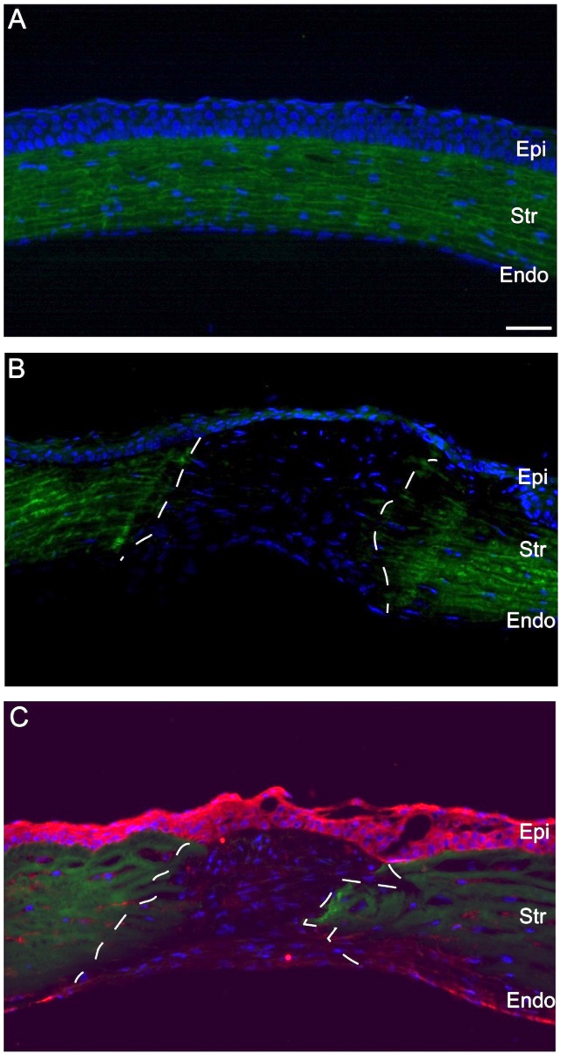

Fig. S1. Keratocan expression is lost following tissue regeneration after injury in normal adult WT corneas. Detection of keratocan in the corneal stroma by fluorescence microscopy using an antibody against keratocan in uninjured adult WT corneas (A). Regenerated stromal matrix 3.5 weeks after full thickness laceration does not express keratocan in normal adult WT mouse (B). Similarly, no eGFP expression by stromal cells was noted in regenerated area in I-*KeramTmG* model 1 month post full thickness laceration. Green stain noted outside regenerated area is DTAF. This chemical -DTAF- was applied to the stroma at the time of injury. DTAF labeled collagen fibrils at the time of injury only and is not present in newly regenerated stroma (C). Bar 50 µm.
